# Cardioprotective effect of nicorandil on isoproterenol induced cardiomyopathy in the Mdx mouse model

**DOI:** 10.1186/s12872-021-02112-4

**Published:** 2021-06-15

**Authors:** Rachel T. Sullivan, Ngoc T. Lam, Margaret Haberman, Margaret J. Beatka, Muhammad Z. Afzal, Michael W. Lawlor, Jennifer L. Strande

**Affiliations:** Medical College of Wisconsin, 8701 W Watertown Plank Rd, Milwaukee, WI 53226 USA

**Keywords:** Duchenne muscular dystrophy, DMD, Cardiomyopathy, Nicorandil

## Abstract

**Background:**

Duchenne muscular dystrophy (DMD) associated cardiomyopathy is a major cause of morbidity and mortality. In an in vitro DMD cardiomyocyte model, nicorandil reversed stress-induced cell injury through multiple pathways implicated in DMD*.* We aimed to test the efficacy of nicorandil on the progression of cardiomyopathy in *mdx* mice following a 10-day treatment protocol.

**Methods:**

A subset of *mdx* mice was subjected to low-dose isoproterenol injections over 5 days to induce a cardiac phenotype and treated with vehicle or nicorandil for 10 days. Baseline and day 10 echocardiograms were obtained to assess cardiac function. At 10 days, cardiac tissue was harvested for further analysis, which included histologic analysis and assessment of oxidative stress. Paired student’s t test was used for in group comparison, and ANOVA was used for multiple group comparisons.

**Results:**

Compared to vehicle treated mice, isoproterenol decreased ejection fraction and fractional shortening on echocardiogram. Nicorandil prevented isoproterenol induced cardiac dysfunction. Isoproterenol increased cardiac fibrosis, which nicorandil prevented. Isoproterenol increased gene expression of NADPH oxidase, which decreased to baseline with nicorandil treatment. Superoxide dismutase 2 protein expression increased in those treated with nicorandil, and xanthine oxidase activity decreased in mice treated with nicorandil during isoproterenol stress compared to all other groups.

**Conclusions:**

In conclusion, nicorandil is cardioprotective in *mdx* mice and warrants continued investigation as a therapy for DMD associated cardiomyopathy.

**Supplementary Information:**

The online version contains supplementary material available at 10.1186/s12872-021-02112-4.

## Introduction

Duchenne muscular dystrophy (DMD) is an X-linked recessive genetic disorder which affects 1:3500–5000 males [1, 2]. DMD is caused by mutations in the dystrophin gene that lead to either complete absence of dystrophin or the expression of a significantly truncated and non-functional protein. Dystrophin, as part of the dystrophin glycoprotein complex, serves important roles in both cell membrane stabilization and cell signaling [3, 4]. Clinically, DMD manifests as progressive loss of muscle function, starting with skeletal muscle weakness with progression to wheelchair dependence beginning in the first decade of life. DMD associated cardiomyopathy becomes more evident starting in the second decade of life. With advances in medical management, including mechanical ventilation, DMD patients are living longer and the cardiomyopathy associated with DMD is becoming a significant cause of morbidity and mortality [5–7]. The mainstay of current medical management of DMD associated cardiomyopathy is the use of generic heart failure medications, including ACE inhibitors and beta blocker therapy [8]. While these strategies afford some cardiac benefit, they do not take into account the specific molecular mechanisms involved in the development of DMD associated cardiomyopathy.

Multiple cellular and metabolic processes have been implicated in the development of DMD associated cardiomyopathy. Contractile properties are abnormal in DMD affected myocardium [9]. Excessive calcium influx into cardiac myocytes has been implicated in cardiac myocyte dysfunction [10, 11]. Additional molecular mechanisms include impaired nitric oxide production, nitric oxide-cyclic GMP pathway dysregulation, increased reactive oxygen species production, and mitochondrial dysfunction [12–14]. Myocardial K_ATP_ channels, which have inherent function in sensing the metabolic state of the cell, have gained attention in recent years as appropriate channel function has been found to have cardioprotective properties during times of metabolic stress [15]. K_ATP_ channels co-localize with dystrophin, and lack of dystrophin leads to inability of K_ATP_ channels to sense intracellular changes in ATP levels [16]. These multiple pathways ultimately culminate to lead to cellular necrosis and fibrosis, which progresses to a clinical phenotype of a dilated cardiomyopathy[17, 18].

Nicorandil is a compound of particular interest as a potential targeted therapy for DMD associated cardiomyopathy, as it modulates several of the metabolic pathways previously shown to be involved in disease development. Nicorandil is a compound with nitrate-like qualities, which acts as a nitric oxide donor, K_ATP_ channel opener, and has anti-oxidant properties [19–21]. Nicorandil has an established pharmacologic safety profile as it is currently in clinical use as an anti-anginal agent and has been shown to be cardioprotective in other forms cardiomyopathy. Cardioprotective effects have been demonstrated in doxorubicin induced cardiomyopathy in both an in vitro cell model and an in vivo rat model [22, 23]. Nicorandil is most well-studied in ischemia–reperfusion injury following acute coronary events. Several animal models, including canine, rabbit, and rat models have demonstrated that pretreatment with nicorandil leads to decreased infarction size in coronary ischemia–reperfusion injury [24–26]. This has translated to clinical benefit in patients, with recent meta-analyses demonstrating benefit of nicorandil administration prior to percutaneous coronary intervention for patients with acute myocardial infarction undergoing primary intervention and patients with coronary artery disease and angina undergoing elective intervention. In both of these groups nicorandil improves ejection fraction and decreases risk of adverse cardiovascular events [27, 28].

For these reasons, it is advantageous to further investigate nicorandil for the treatment of DMD associated cardiomyopathy as it can be quickly translated into a feasible treatment option for these patients. Our lab was the first to study nicorandil in an in vitro DMD model by studying its effect in dystrophin-deficient induced pluripotent stem cell (iPSC) derived cardiomyocytes from DMD patients. Our study demonstrated that in the presence of a cellular stressor, nicorandil was protective against cardiomyocyte injury by decreasing cell injury and cell death through mechanisms that involved preventing the stress-induced increase in reactive oxygen species and loss of the mitochondrial membrane potential [29]. Additionally, we previously showed protective benefits of nicorandil in isolated perfused *mdx* hearts subject to global ischemia and recovery [29]. The purpose of this study is to extend the investigation of nicorandil to determine whether it would maintain cardioprotective effects in the *mdx* mouse heart in vivo.

## Methods

### Animals

C57BL/10ScSn-Dmd^mdx^/J (*mdx*) mice were purchased from the Jackson Laboratory (Bar Harbor, Maine) at 8–10 weeks of age then acclimated until the desired age to undergo the subsequently described experimental protocol. A total of 48 female mice (10–14/group) were used for this study. Female mice were used in our study based on prior research demonstrating a more robust cardiac phenotype in *mdx* females compared to males [30]. Animal group size was determined empirically, with the aim to minimize animal numbers during this early-stage in vivo study. Animals were randomized per cage, with all in the same cage receiving the same treatment. Investigators were not blinded to treatment group allocation. Housing and procedure rooms were under specific pathogen-free conditions. The mice had a 12-h day/night cycle, with daytime being from 7 am to 7 pm. Mice were fed a standard diet of Purina irradiated 5LOD. All animals in this study received humane care in compliance with the “Guide for the Care and Use of Laboratory Animals” published by the US National Institutes of Health (NIH Publication No. 85-23, revised 1996). All animal experiments were performed at the Medical College of Wisconsin and were approved by the institutional animal care and use committee (procedure protocol AUA00003547). This study involved no human subject research.

### Study protocol

**Fig. 1:**
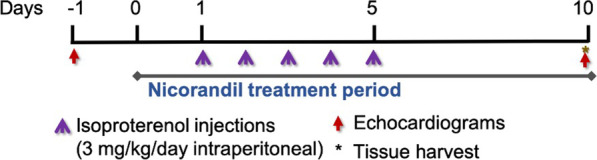
Schematic of the 10 day treatment protocol

A short-term subacute injury model using low-dose isoproterenol (Sigma, St. Louis, Missouri) was utilized to induce a cardiac phenotype in 11–14-week mice. The isoproterenol (3 mg/kg) was administered intraperitoneally once daily in the early afternoon for the first 5 days of the ten-day treatment protocol. This dose was chosen empirically with the goal to induce mild cardiac injury, while avoiding mortality. Isoproterenol injury is a well-described means of inducing cardiac injury in *mdx* mice by means of increased oxidative stress, and sarcolemmal damage [31]. Dosing, mode of isoproterenol delivery, and mortality vary considerably between studies using an isoproterenol induced cardiac phenotype [32, 33]. While beta receptor agonism with isoproterenol has been shown to lead to cardiac hypertrophy in normal myocardium, this would not be expected with beta agonism in dystrophin deficient myocardium given its well-described phenotype of a dilated cardiomyopathy without pathologic cardiac hypertrophy [34–36]. Nicorandil (Selleckchem, Houston, Texas) was given via drinking water at 6 mg/kg/day for the duration of the ten-day treatment protocol [37, 38]. The vehicle was ddH20, which was administered in the drinking water in the same period as nicorandil. It is not expected to have any effects. The animals were assigned to 4 groups: vehicle (n = 10), nicorandil treatment (n = 10), isoproterenol treatment (n = 14), and isoproterenol plus nicorandil treatment (n = 14). At the ten-day timepoint, the hearts were harvested, and blood was collected for further analysis. Study protocol is outlined in Fig. 1.

### Echocardiography and image analysis

Transthoracic echocardiograms were performed in anesthetized (2.5% isoflurane) mice at baseline and at 10 days. Focused 2-dimensional echocardiogram with Doppler was performed using a commercially available echocardiographic system (Vivid 7, General Electric) with an 11 MHz M12-L linear array transducer. An M-mode image was taken in the cardiac short axis at the level of the papillary muscles. From this image the following measurements were obtained: ejection fraction % (EF), fractional shortening % (FS), left ventricular end diastolic volume (LVEDV), and left ventricular end systolic volume (LVESV). An average of three consecutive cardiac cycles was used for each measurement.

### Histology

The heart was dissected and fixed in 4% paraformaldehyde overnight at 4 °C. The tissues were then washed with PBS and embedded using sucrose and OCT. Frozen hearts were sectioned onto slides in 8 micron thick axial sections. Slides were stained with Masson’s Trichrome stain and hematoxylin counterstain (Masson Trichrome Special Stain Kit, Leica, Buffalo Grove, Illinois). Images of the entire section were obtained (Nikon Super Coolscan 9000). Metamorph image analysis software was used to quantify percent fibrosis by means of thresholding the specific blue coloration of collagen positive regions. The thresholded area was compared to total area of cardiac tissue and represented as percentage of total tissue area. An average of two sections for each sample was used for each measurement.

### Protein extraction and western blot

Protein extraction and immunoblot analysis was performed on individual mouse heart protein isolates as previously described (n = 2 per group) [39]. Proteins were isolated by homogenization with a Dounce homogenizer in RIPA buffer (Sigma, St. Louis, Missouri) and Halt protease/phosphatase inhibitor (ThermoFisher, Waltham, Massachusetts) and centrifuged at 11,000*g* at 4 °C for 10 min. Protein concentration was measured by commercially available bicinchoninic acid assay kit (Thermo Scientific, Waltham, Massachusetts). Fifteen micrograms of protein per sample was separated on a 4–20% Criterion TGX Stain-Free Protein Gel (BioRad, Hercules, California) and transferred onto a polyvinylidene difluoride membrane. After the membrane was blocked with SuperBlock T20 (TBS) Blocking Buffer (ThermoFisher, Waltham, Massachusetts) with 0.1% Tween-20, the membrane was incubated with primary antibody Anti-NADPH oxidase 4, 1:300 dilution (ab133303, Abcam, Cambridge, MA), Anti-SIRT3, 1:1,000 dilution (ab264041, Abcam, Cambridge, MA), Anti-TNF alpha, 1:1,000 dilution (ab6671, Abcam, Cambridge, MA), Anti-NF-kB p65, 1:2,000 dilution (ab16502, Abcam, Cambridge, MA), anti-SOD2/MnSOD, 1:5,000 (ab13533, Abcam, Cambridge, MA) and Vinculin (E1E9V) XP Rabbit mAb (HRP Conjugate), 1:5,000 dilution (18799, Cell Signaling, Danvers, MA) for 2 h at room temperature. After washing, the blots were incubated with a secondary antibody at a 1:10,000 dilution (donkey anti-rabbit peroxidase conjugated, 711-035-152, Jackson ImmunoResearch, West Grove, PA) for 1 h at room temperature followed by chemiluminescence solution (Amersham ECL Prime, RPN2236, Cytiva, Marlborough, MA) prior to imaging on a ChemiDoc imaging system (BioRad, Hercules, California). Protein quantification was performed using BioRad ImageLab software and compared to the vinculin loading control.

### Xanthine oxidase activity

Xanthine oxidase activity was determined in mouse serum (n = 4 per group) using a commercially available fluorometric assay kit (Cayman Chemical, Ann Arbor, Michigan).

### Reverse transcriptase PCR

RNA isolation was performed according to previously described protocol [29]. The forward and reverse primer sequences of following primers were used: superoxide dismutase 2 (SOD2) exon 3 to 4 (forward 5′-TCAATGGTGGGGGACATATT-3′, reverse 5′-GAACCTTGGACTCCCACAGA-3′), sirtuin 3 exon 4–5 (forward 5′-ACTTCCTGAGGCTCCTCCAC-3′, reverse 5′- ACAGACCGTGCATGTAGCTG-3′), NADPH oxidase (NOX)-2 (forward 5′-TCGAAAACTCCTTGGGTCAG-3′, reverse 5′-AGCATTTGGTGTGATGGTCA-3′), NOX-4 (forward 5′-AGCATCTGCATCTGTCCTGA-3′, reverse 5-CATGTGATGTGTAGAGTCTTGC-3′). Mouse GAPDH was used as a normalization control. Experiments were performed with an n = 6 per group, with the exception of the vehicle treated group given limited tissue availability.

### Statistical analysis

Statistical analyses were performed using GraphPad Prism 9 software (Version 9.1.0). Data are displayed as mean ± standard error of the mean. Paired student’s t test was used for in group comparisons between baseline and day 10 echocardiographic parameters. For the remainder of experimental assays, one-way ANOVA with Tukey’s multiple comparisons test post-hoc analysis was used for multiple group comparisons. P values of less than 0.05 were considered significant.

## Results

### Nicorandil is protective against isoproterenol-induced cardiac injury

Previously, our lab demonstrated that treatment with nicorandil conferred protective effects in iPSC derived cardiomyocytes from DMD patients by means of decreasing cell injury and death, maintenance of the mitochondrial membrane potential, and decreasing reactive oxygen species production [29]. To assess the effect of nicorandil in vivo*,* noninvasive functional cardiac assessment was performed via transthoracic echocardiograms both at baseline and after a 10 day treatment protocol in 11–14 week old *mdx* mice. As part of this treatment protocol, a subset was treated with once daily intraperitoneal injections of isoproterenol for 5 days to induce a mild cardiac phenotype. We intentionally aimed for a mild cardiac injury so as to not induce death in treated mice in order to maintain the ability for in vivo functional cardiac assessment. Additionally, we felt that a mild cardiac injury in our induced cardiac phenotype was more analogous to disease progression of DMD associated cardiomyopathy in humans, which presents as gradual decline in cardiac function rather than an acute, severe decline in function. Comparisons were made between baseline and day 10 measurements within each group. Baseline measurements of ejection fraction (EF), fractional shortening (FS), left ventricular end systolic volume (LVESV), left ventricular end diastolic volume (LVEDV), and stroke volume (SV) were comparable between groups. Isoproterenol induced a mild cardiac injury with a small, but statistically significant decrease in cardiac function as indicated by a decreased EF and FS and an increased LVESV. Concomitant treatment with nicorandil prevented isoproterenol-induced cardiac injury (Table 1). Additionally, isoproterenol induced a cardiac phenotype of a dilated cardiomyopathy in isoproterenol treated animals, with increased LVEDV and increased stroke volume, which is consisted with the dilated cardiomyopathy phenotype in DMD patients. Nicorandil prevented left ventricular dilation (Table 1). Heart rate was uniformly higher in the day 10 measurements, statistically significantly so in the isoproterenol treated group. This likely was more representative of level of sedation given the presence of higher heart rate at day 10 measurement in all groups, though this may have been more significant in the isoproterenol treated group due to the development of the induced cardiomyopathy.

**Table 1:** Demographic characteristics and echocardiographic data

Mdx mouse groups:	Vehicle (n = 10)	Nicorandil (n = 10)	Isoproterenol (n = 14)	Nicorandil + Isoproterenol (n = 14)
*Baseline characteristics*				
Age at study end (weeks)	13.0 ± 0.52	13.4 ± 0.44	13.4 ± 0.47	13.4 ± 0.51
Body weight at study end (g)	25.8 ± 0.53	26.2 ± 0.45	26.4 ± 0.46	25.1 ± 0.45
*Echocardiographic measurements*				
Heart rate (beats per minute)				
Baseline	419 ± 16	472 ± 16	394 ± 19	451 ± 14
Day 10	501 ± 11*	485 ± 14	454 ± 14*	489 ± 13*
Ejection fraction (%)				
Baseline	71.3 ± 1.8	75.9 ± 2.1	75.2 ± 1.6	75.9 ± 1.6
Day 10	71.9 ± 2.3	72.6 ± 2.5	68.1 ± 2.6*	74.8 ± 1.6
Fractional shortening (%)				
Baseline	35.3 ± 1.5	39.2 ± 1.8	38.6 ± 1.3	39.2 ± 1.4
Day 10	36.0 ± 1.8	36.6 ± 2.0	33.4 ± 1.9*	38.2 ± 1.5
Left ventricular end systolic volume (mL)				
Baseline	0.030 ± 0.003	0.027 ± 0.004	0.028 ± 0.003	0.025 ± 0.003
Day 10	0.040 ± 0.007	0.040 ± 0.005	0.047 ± 0.005*	0.030 ± 0.003*
Left ventricular end diastolic volume (mL)				
Baseline	0.116 ± 0.008	0.190 ± 0.011	0.111 ± 0.005	0.099 ± 0.007
Day 10	0.119 ± 0.015	0.128 ± 0.011	0.144 ± 0.007*	0.114 ± 0.009
Stroke volume (mL)				
Baseline	0.083 ± 0.006	0.080 ± 0.007	0.083 ± 0.003	0.074 ± 0.005
Day 10	0.083 ± 0.008	0.093 ± 0.008	0.096 ± 0.005*	0.082 ± 0.007

Echocardiograms were performed at baseline and at 10 days. Isoproterenol induced left ventricular dilation and a mild cardiac injury with a decrease in ejection fraction and fractional shortening and an increased end systolic volume. Nicorandil was protective against left ventricular dilation and isoproterenol-induced injury. (*p < 0.05 vs same group baseline measurement)

### Nicorandil is protective against isoproterenol-induced cardiac fibrosis

**Fig. 2:**
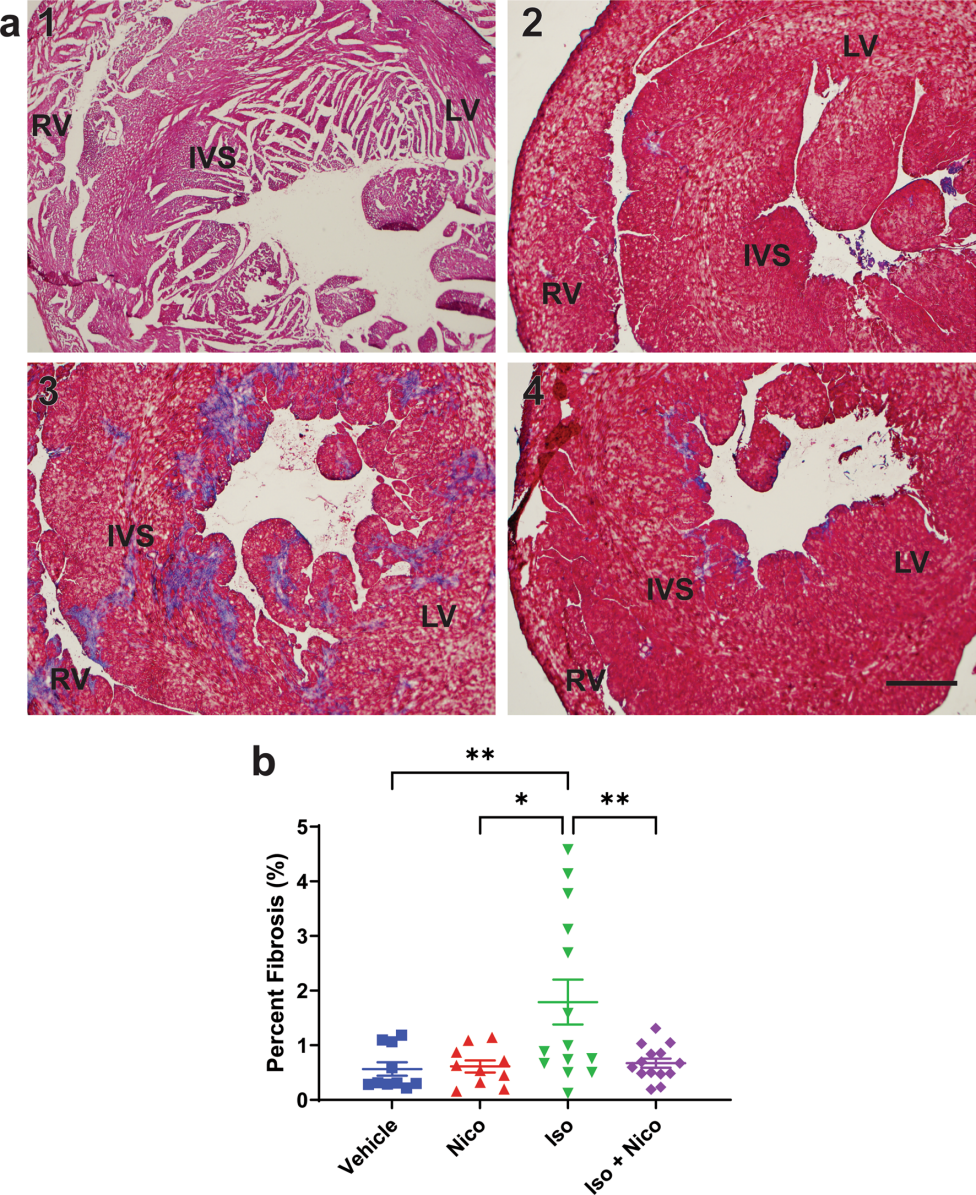
Histologic assessment of myocardial fibrosis. Frozen mouse heart tissue was sectioned and stained with Masson’s trichrome stain to assess myocardial fibrosis (blue tissue coloration). **a** Representative images of Masson’s trichrome stained heart tissue, with groups identified as follows: 1: Vehicle, 2: Nicorandil, 3: Isoproterenol, 4: Isoproterenol + Nicorandil. Images are centered in the interventricular septum (IVS) and areas corresponding to right ventricle (RV) and left ventricle (LV) are labeled. **b** Quantification of percent fibrosis on Masson’s trichrome staining, represented as percentage of total tissue area ± SEM. Isoproterenol led to an increase in percent fibrosis. Nicorandil was protective against isoproterenol-induced fibrosis. Number of samples for each experimental group: vehicle n = 10, Nicorandil n = 10, Isoproterenol n = 14, Isoproterenol + Nicorandil n = 14. SEM, standard error of the mean. (*p < 0.05 vs vehicle)

Frozen cardiac tissue sections were evaluated for collagen deposition, which is representative of myocardial fibrosis. Tissue sections from all study mice were stained with Masson’s trichrome stain, which enables one to distinguish blue stained collagen fibers from red stained muscle fibers. As shown in Fig. 2, vehicle and nicorandil-only treated hearts have minimal fibrosis with 0.6–0.7% of total cardiac tissue area consisting of collagen. Isoproterenol induced a significant degree of fibrosis compared to baseline with a nearly three-fold increase in collagen staining at 1.8 ± 0.4% of total cardiac tissue area consisting of collagen. Concomitant treatment with nicorandil prevented the development of cardiac fibrosis, with those mice receiving both nicorandil and isoproterenol injury protocol having comparable percentage fibrosis compared to vehicle treated animals.

### Nicorandil modulates reactive oxygen species formation

**Fig. 3:**
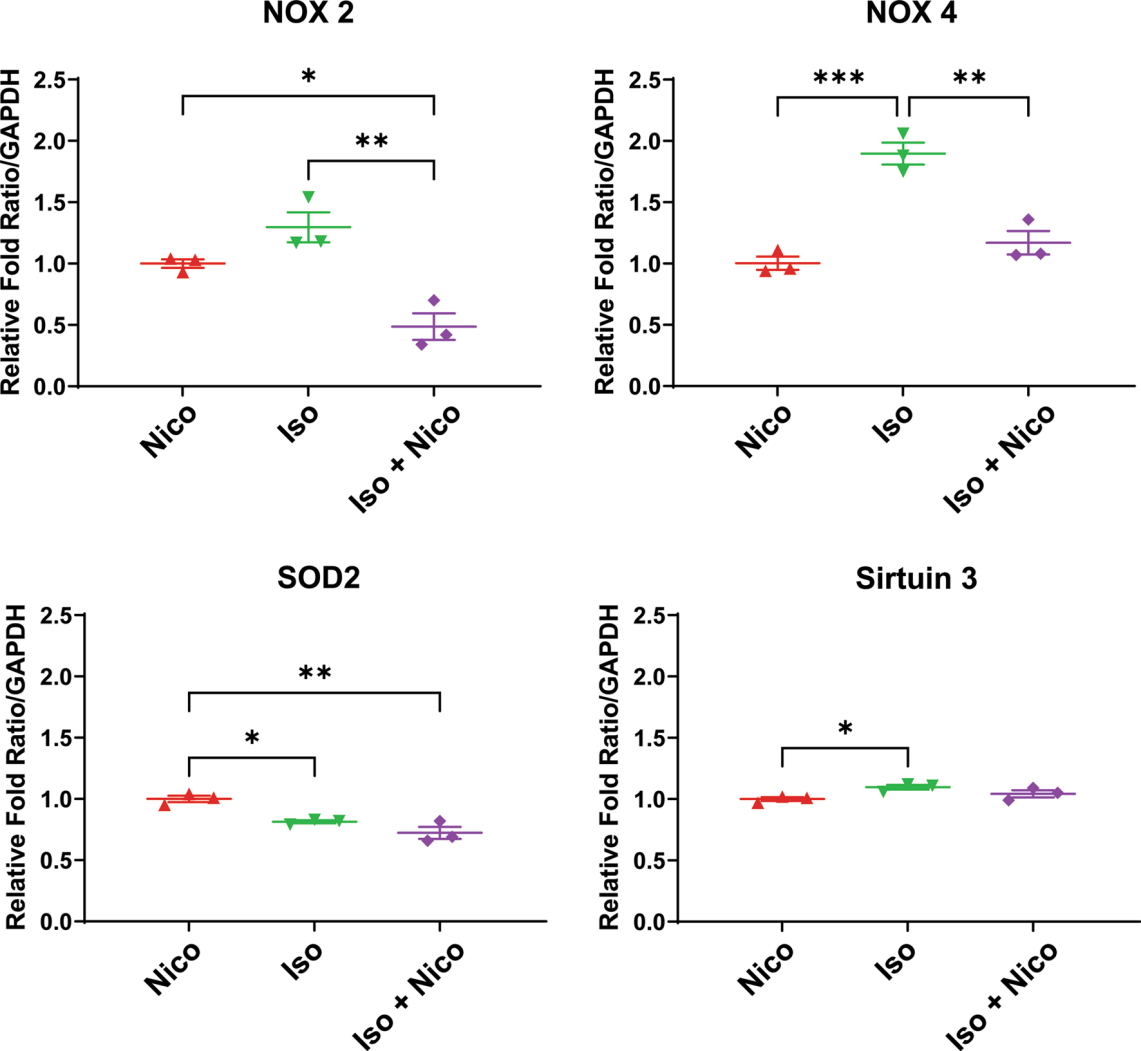
Reactive oxygen species gene expression. Quantitative RT-PCR was performed on mouse cardiac tissue to quantify mRNA expression of select genes involved in reactive oxygen species formation, including NOX2, NOX4, SOD2, and Sirtuin 3. Expression of NADPH Oxidase (NOX) 4 and NOX 2 were increased with isoproterenol stress. Treatment with nicorandil prevented this change in gene expression. (n = 6, *p ≤ 0.05, **p ≤ 0.01, and ***p ≤ 0.001)

**Fig. 4:**
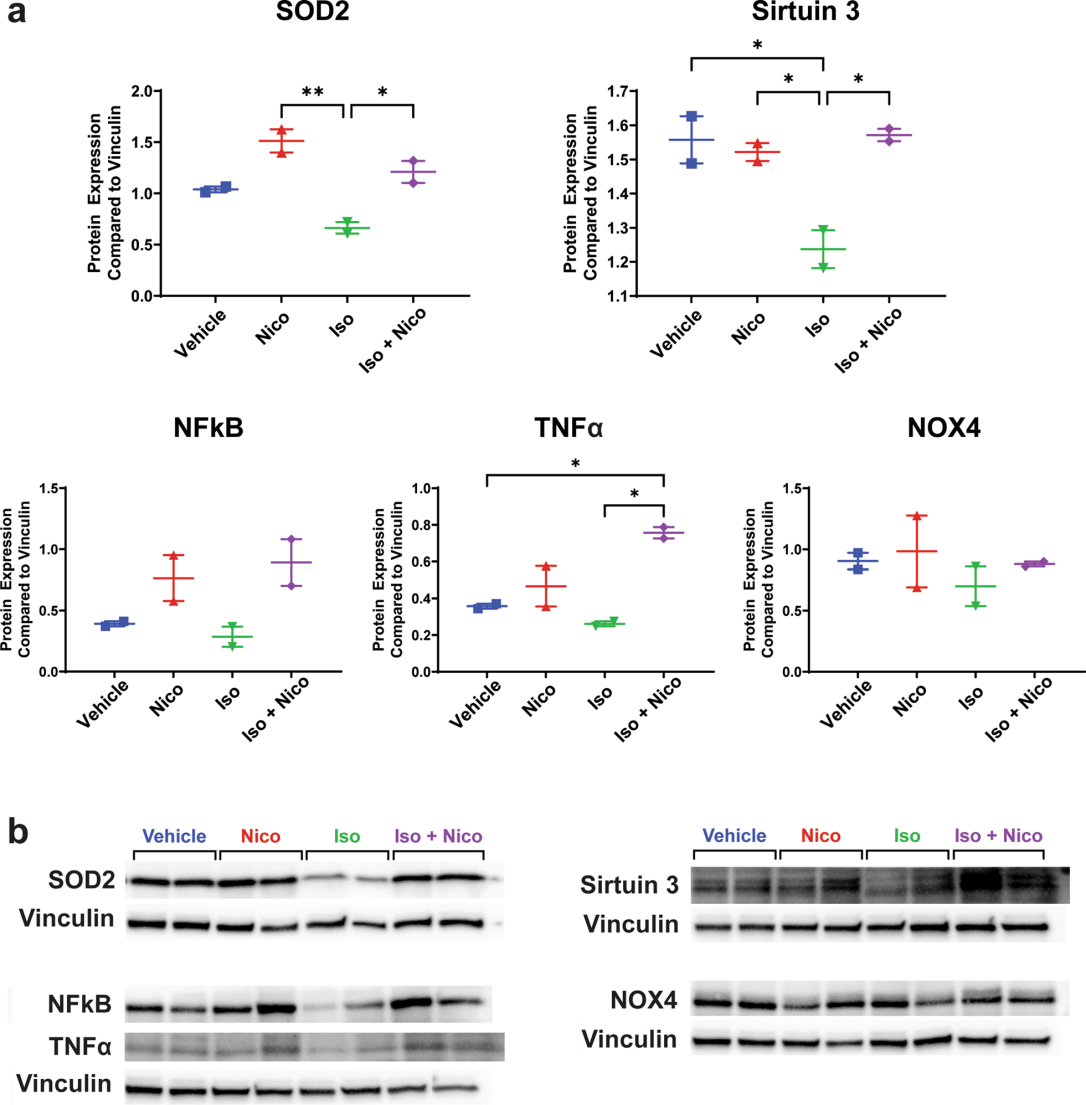
Reactive oxygen species protein expression. Western blot was performed on mouse cardiac tissue to assess protein expression of selected proteins involved in reactive oxygen species production and inflammation. Relative protein expression is shown in part **a**, and cropped representative immunoblot images are shown in part **b**. the vinculin loading control is shown for each separate western blot that is shown in the figure. Note that NFkB and TNFα were assessed using different regions of the same western blot membrane. Full-length blots are presented in Additional files 1, 2, 3, 4: Figs. 1–4 (n = 2, *p ≤ 0.05, **p ≤ 0.01)

It is well-established that increased reactive oxygen species (ROS) production plays a role in many cardiac diseases, including in the development of DMD associated cardiomyopathy. Quantitative RT-PCR was performed to assess gene expression of several genes known to be involved in ROS production. Of note, for our RT-PCR experiments, Nicorandil-only treated *mdx* mice were used as a control due to tissue availability. This group was designed to act as a functional secondary control since mice of this age would not be expected to naturally exhibit a cardiac phenotype without employing means to induce cardiac involvement. NADPH oxidase (NOX) is an enzyme which functions in the creation of superoxide free radicals. There are several NOX isoforms, with the two most expressed in cardiac tissue being isoforms NOX-2 and NOX-4 [40]. We found that expression of NOX-4 was significantly increased with isoproterenol compared to nicorandil only treated animals. There was a similar, though not statistically significant, increase in NOX-2 gene expression with isoproterenol. Treatment with nicorandil prevented increased NOX-2 and NOX-4 expression while undergoing isoproterenol stress (Fig. 3). NOX-4 protein expression did not differ significantly between groups (Fig. 4).

Nicorandil decreases mitochondrial ROS levels by increasing antioxidant gene expression, such as SOD2 and sirtuin 3 [41–43]. In our previous in vitro studies, we showed that nicorandil increased SOD2 gene expression after stress which was associated with a decrease in mitochondrial ROS levels as well [29]. Examining the myocardium from each group did not reveal a significant change in SOD2 or sirtuin 3 gene expression (Fig. 3). However, SOD2 protein expression increased significantly with nicorandil treatment, irrespective of concomitant isoproterenol stress (Fig. 4). Sirtuin 3 protein expression did not differ between groups (Fig. 4).

Previous studies have demonstrated that the inflammatory markers, nuclear factor kappa beta (NF-kB) and tumor necrosis factor alpha (TNF-α) are increased in *mdx* myocardium[44]. To evaluate the impact of nicorandil on inflammation, protein expression of NFkB and TNF-α was evaluated. There was no difference in NF-kB protein expression between groups. TNF-α protein expression was increased in those treated with concurrent isoproterenol and nicorandil compared to both vehicle and isoproterenol treated animals (Fig. 4). Notably, there was no inflammatory cell infiltrate seen in the histologic sections from any group (Fig. 2).

**Fig. 5:**
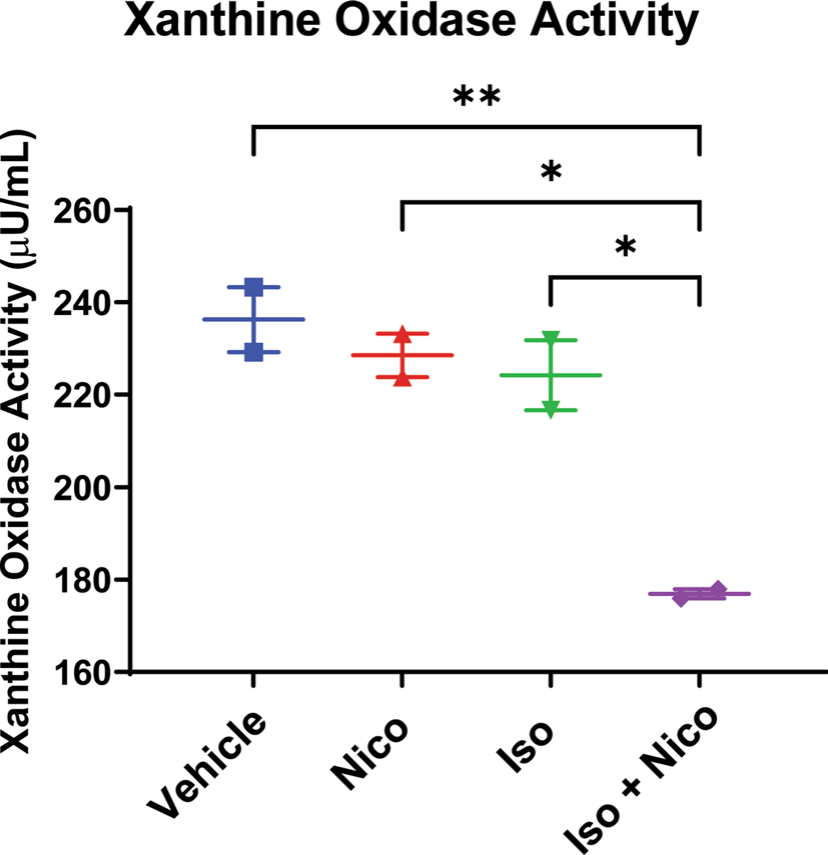
Xanthine oxidase activity: Xanthine oxidase activity was assessed in cardiac tissue by fluorometric assay kit (Cayman Chemical). Nicorandil led to a decrease in xanthine oxidase activity with concomitant isoproterenol treatment protocol (n = 4, *p < 0.05 vs vehicle)

Nicorandil has also been shown to decrease ROS by inhibiting xanthine oxidase activity [45]. Furthermore, xanthine oxidative inhibitors decreased ROS levels in DMD-iPSC derived cardiomyocytes suggesting that xanthine oxidase contributes to the oxidative stress in DMD associated cardiomyopathy [29]. Therefore, we evaluated whether nicorandil was able to alter xanthine oxidase activity using a commercially available fluorometric assay kit. Xanthine oxidase activity was significantly decreased in those *mdx* mice treated with nicorandil while undergoing isoproterenol stress compared to all other treatment groups (Fig. 5).

## Discussion

This study provides the first evidence that nicorandil exerts cardioprotective effects against DMD associated cardiomyopathy in *mdx* mice in vivo. We demonstrated that nicorandil prevents isoproterenol induced left ventricular dilation, cardiac dysfunction and cardiac fibrosis. An important measure by which prior investigators have demonstrated the cardioprotective effect afforded by nicorandil in other disease states is by means of decreased ROS production. Nicorandil has been found to decrease ROS produced from NADPH oxidase and xanthine oxidase and to increase anti-oxidants such as SOD2, glutathione peroxidase, and catalase [21–23, 29]. Our study further supported the ROS modulating effect of nicorandil. Nicorandil mitigated isoproterenol-induced increase in NADPH oxidase gene expression, increased SOD2 protein expression, and decreased xanthine oxidase activity. Our lab previously demonstrated in an in vitro DMD model that nicorandil was protective against cardiomyocyte injury and showed protective benefits in the isolated perfused *mdx* heart [29]. This study expands on that work and gives supportive evidence to warrant continued investigation of nicorandil as a treatment of DMD associated cardiomyopathy.

The current standard of care for management of DMD associated cardiomyopathy includes the use of generic heart failure therapies with ACE inhibitors, angiotensin receptor blockers, and beta blocker therapies based on few small, clinical studies demonstrating clinical benefit on left ventricular function and survival [46–49]. There is, however, a notable lack of therapies directed toward the specific cellular mechanisms that lead to DMD associated cardiomyopathy. Significant attention in recent years has been paid to gene targeted therapies, which are in varying stages of development [50]. These technologies may also be mutation-specific and may have differential effectiveness in cardiac versus skeletal muscle, which could limit the timely and broad applicability. Nicorandil is of particular interest as a targeted therapeutic given that its multiple mechanisms of action as a nitric oxide donor, anti-oxidant, and K_ATP_ channel opener allow it to act on many of the molecular processes involved in the development of DMD associated cardiomyopathy [19–21]. Compounds that target just one of these molecular pathways have been investigated without significant success in translating to broad clinical applicability. For instance, to combat the abnormal nitric oxide-cyclic GMP signaling in DMD associated cardiomyopathy, phosphodiesterase (PDE) 5 inhibitors have been explored [51]. Sildenafil prevented the diastolic dysfunction seen echocardiographically in 15 month old *mdx* mice. Further, when started after the onset of cardiac dysfunction, sildenafil reversed these findings of cardiomyopathy. These encouraging findings were unfortunately not translated into clinical benefit with no improvement in cardiac magnetic imaging derived indices of cardiomyopathy in adults with either Duchenne or Becker muscular dystrophy [52, 53]. Affecting only one of the multiple molecular processes involved in the pathogenesis of DMD associated cardiomyopathy with PDE 5 inhibition may not have been enough to prevent the development of cardiomyopathy.

The oxidative stress etiology of the development of DMD associated cardiomyopathy has also been explored as a potential modifiable target in pre-clinical studies. NOX-2 inhibition with the compound VAS2870 improved contractility and calcium handling in isolated *mdx* cardiomyocytes, however NOX inhibition has not yet been explored in an in vivo model [12]. Resveratrol is a polyphenol that activates the NAD + -dependent protein/histone deacetylase SIRT1, which has been found to play a regulatory role in cell survival and stress response. Resveratrol has gained interest as a potential therapeutic in DMD and showed improved skeletal muscle phenotypes in *mdx* mice along with decreased indices of oxidative stress [54]. Resveratrol has also demonstrated cardioprotective effects in *mdx* mice, with prevention of cardiac hypertrophy and diastolic dysfunction, improved fractional shortening on echocardiogram, and decreased fibrosis [55, 56]. These improved cardiac measures were accompanied by evidence of improved markers of damaged mitochondrial DNA and decreased ROS levels. Mixed results have been seen when assessing modulators of systemic inflammation. Systolic and diastolic dysfunction as measured by direct invasive measures was prevented in *mdx* mice treated for a period of time with the transforming growth factor-ß inhibitor, pirfenidone, until 15 months of age. However, pirfenidone did not afford the anticipated improvement in cardiac fibrosis, given its previously demonstrated anti-fibrotic effect in other organs [57]. Conversely, the tumor necrosis factor blocker, Remicade, did prevent the development of fibrosis in both skeletal and cardiac muscle, though was associated with decreased left ventricular ejection fraction by echocardiogram after 32 weeks of treatment [58]. Considering nicorandil targets three separate involved molecular pathways, it may possess greater therapeutic potential and may be superior to other therapeutic candidates.

Our study is not the first to speculate that a more effective therapeutic regimen may be one that acts on multiple pathways implicated in the development of DMD associated cardiomyopathy. One group analyzed the effect of co-administration of ibuprofen and isosorbide dinitrate in *mdx* mice until ten to eleven months of age. Treatment with this combination of medications had cardioprotective effect with preservation of left ventricular thickness, decreased fibrosis and myocardial inflammation, and improved cardiac responsiveness to dobutamine stress [59]. Treatment with naproxinod, which is a nitric oxide donating derivative of naproxen, also demonstrated cardioprotective effect. Treatment with naproxinod through 9 months of age prevented a decrease in ejection fraction and fractional shortening on echocardiogram and demonstrated a trend in decreased fibrosis on histologic analysis [60]. K_ATP_ channel openers have not previously been assessed for effectiveness in pre-clinical or clinical in vivo models of DMD. Given the clinical effectiveness of K_ATP_ channel openers in ischemia reperfusion injury and other forms of cardiomyopathy, we are optimistic that the synergistic effect of K_ATP_ opening in addition to both anti-inflammatory and nitric oxide donating properties will be a beneficial combination for the treatment of DMD associated cardiomyopathy.

We do recognize that our study does have limitations. This study was performed in young *mdx* mice subjected to low dose isoproterenol to induce cardiac stress. This subacute injury mouse model simulates the typical clinical course seen in DMD patients who often present with subclinical cardiac fibrosis and borderline or mild decreases in cardiac function. In this subacute injury model, the echocardiographic evidence of cardiac injury is mild, yet a significant increase in myocardial fibrosis accompanied this change in cardiac function associated with isoproterenol injury protocol. The degree of isoproterenol induced fibrosis was more striking, which mimics the clinical course of cardiomyopathy in humans in which fibrosis can be detected by cardiac magnetic resonance imaging prior to the onset of cardiac dysfunction [61]. Further, there was significant variability in degree of isoproterenol induced cardiac injury. All mice, including those with minimal cardiac injury, were included in final analyses, which if anything would underestimate the benefits of nicorandil in our study.

We demonstrated modulation of ROS pathways, though further work needs to be done to better clarify the specific mechanisms by which ROS modulation is occurring. NOX expression showed no notable change in protein expression by immunoblot, which differed from our RT-PCR findings. Similarly, SOD2 showed no notable change in gene expression by RT-PCR, which differed from our immunoblot findings. Interestingly, xanthine oxidase showed no change in expression/activity from untreated vehicle with isoproterenol stress. Rather, nicorandil’s benefits were only seen when given with concomitant isoproterenol stress. This phenomenon has not been seen in other mechanistic studies on nicorandil and would require further investigation to elucidate the mechanism behind these findings. We hypothesize that nicorandil’s modulation of ROS production may not be appreciable until given in conjunction with an ROS producing stressor. If this were the case, nicorandil-mediated decrease in XO mediated superoxide creation would have beneficial physiologic effects with decreased ROS burden. Notably, the isoproterenol induced stressor concluded 5 days prior to tissue collection. While our experimental timing afforded the opportunity to allow for recovery with continued nicorandil treatment, we may have missed the peak inflammatory response to isoproterenol administration and, accordingly, the ability to speculate clearly on mechanisms of reactive oxygen species modulation by nicorandil treatment. Our findings do, however, overall demonstrate that reactive oxygen species production is clearly affected by nicorandil administration, with an overall decrease in reactive oxygen species production.

## Conclusions

In conclusion, we report that nicorandil exerts cardioprotective effects in an in vivo* mdx* mouse model with a prevention of isoproterenol-mediated cardiac dysfunction and fibrosis and anti-oxidant promoting effects seen on many mediators of ROS production. Nicorandil has three mechanisms of action, being a nitric oxide donor, an anti-oxidant, and K_ATP_ channel opener. We speculate that the combined action on these separate implicated pathways will afford greater potential as a targeted therapeutic for DMD associated cardiomyopathy. Ongoing studies include a long-term natural history study exploring the cardioprotective effectiveness of nicorandil in the naturally occurring cardiac phenotype in *mdx* mice before the longer term goal to transition into human clinical trials.

## Supplementary Information

**Additional file 1**. This supplemental figure shows the uncropped original blots for vinculin loading control and superoxide dismutase 2 (SOD2). 

**Additional file 2**. This supplemental figure shows the uncropped original blots for vinculin loading control and sirtuin 3. The sirtuin 3 band of interest is identified by the red arrow. 

**Additional file 3**.This supplemental figure shows the uncropped original blots for vinculin loading control, NFkB, and TNFα. These were assessed on different regions of the same membrane. The NFkB and TNFα bands of interest are identified by the red arrows.

**Additional file 4**.This supplemental figure shows the uncropped original blots for vinculin loading control and NADPH oxidase 4 (NOX4).

## Data Availability

The datasets used and/or analysed during the current study are available from the corresponding author on reasonable request.

## Abbreviations

DMD: Duchenne muscular dystrophy
EF: Ejection fraction
FS: Fractional shortening
LVEDV: Left ventricular end diastolic volume
NOX: NADPH oxidase
iPSC: Induced pluripotent stem cell
SOD2: Superoxide dismutase 2
ROS: Reactive oxygen species
cMRI: Cardiac magnetic resonance imaging
BMD: Becker muscular dystrophy
PDE: Phosphodiesterase
