# Supplementary figures and images for: Cardioprotective effect of nicorandil on isoproterenol induced cardiomyopathy in the Mdx mouse model
Source: BMC Cardiovasc Disord. 2021 Jun 15;21:302. doi: 10.1186/s12872-021-02112-4 (PMC8207777; doi:10.1186/s12872-021-02112-4)

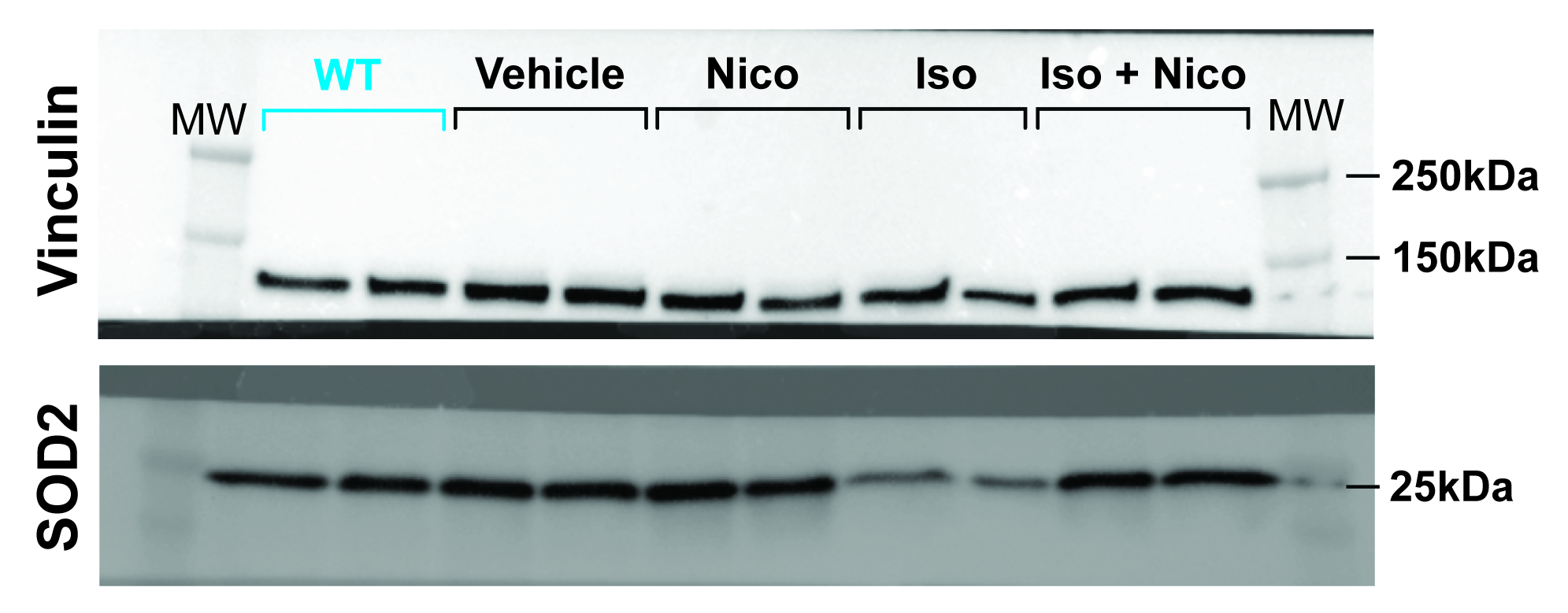

Supplement: Supplementary file 1 — Additional file 1. This supplemental figure shows the uncropped original blots for vinculin loading control and superoxide dismutase 2 (SOD2). [file 12872_2021_2112_MOESM1_ESM.tif]

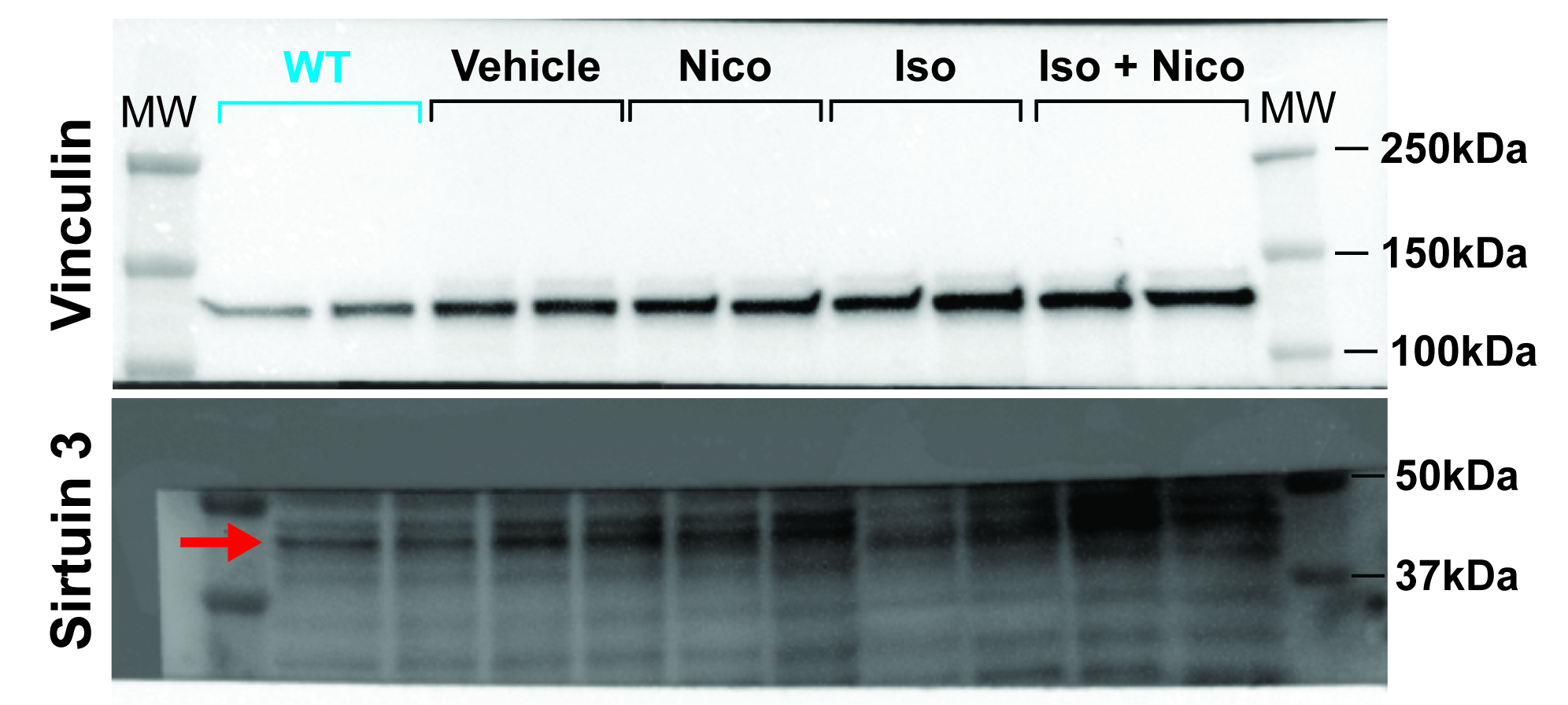

Supplement: Supplementary file 2 — Additional file 2. This supplemental figure shows the uncropped original blots for vinculin loading control and sirtuin 3. The sirtuin 3 band of interest is identified by the red arrow. [file 12872_2021_2112_MOESM2_ESM.tif]

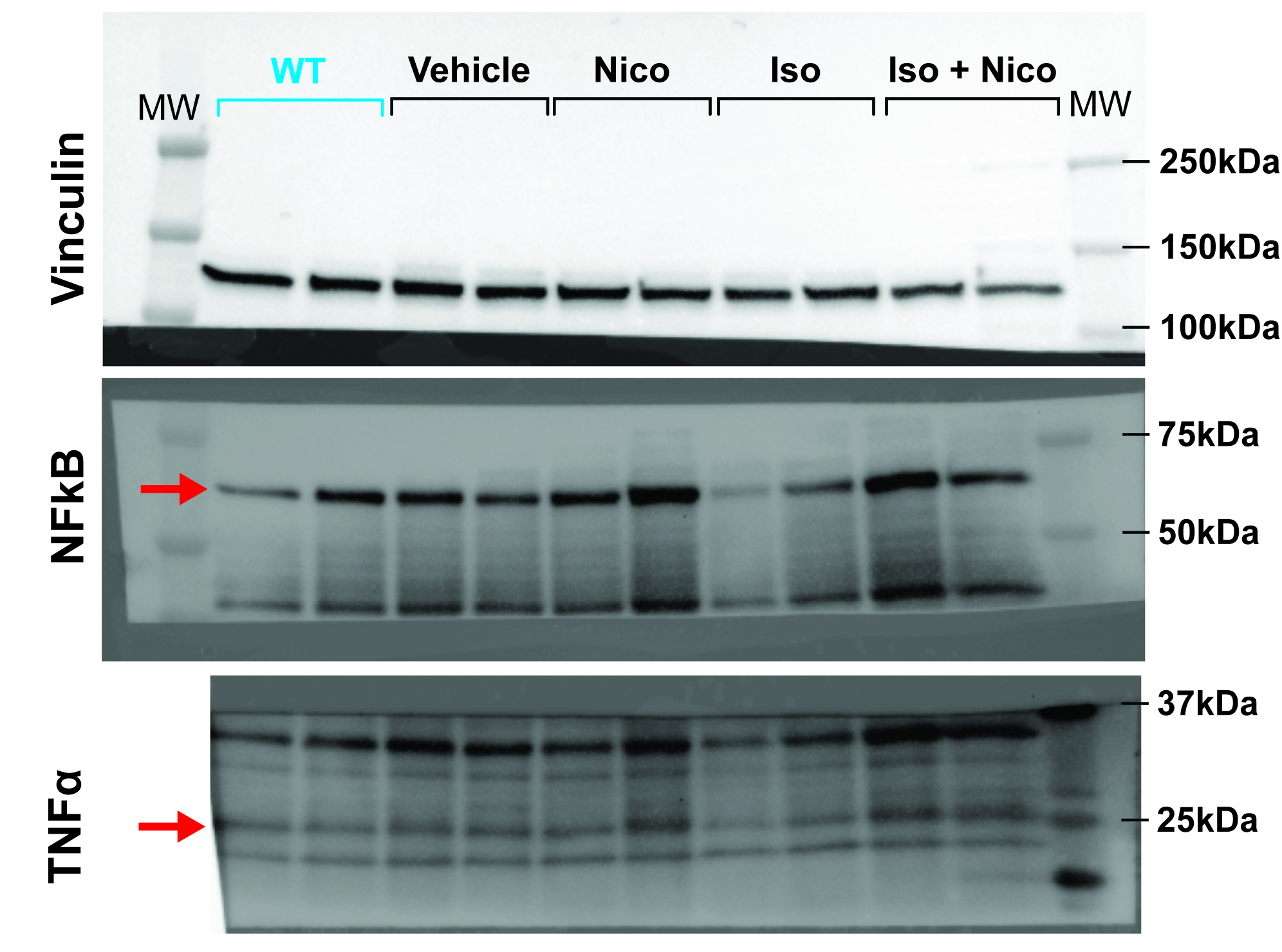

Supplement: Supplementary file 3 — Additional file 3.This supplemental figure shows the uncropped original blots for vinculin loading control, NFkB, and TNFα. These were assessed on different regions of the same membrane. The NFkB and TNFα bands of interest are identified by the red arrows. [file 12872_2021_2112_MOESM3_ESM.tif]

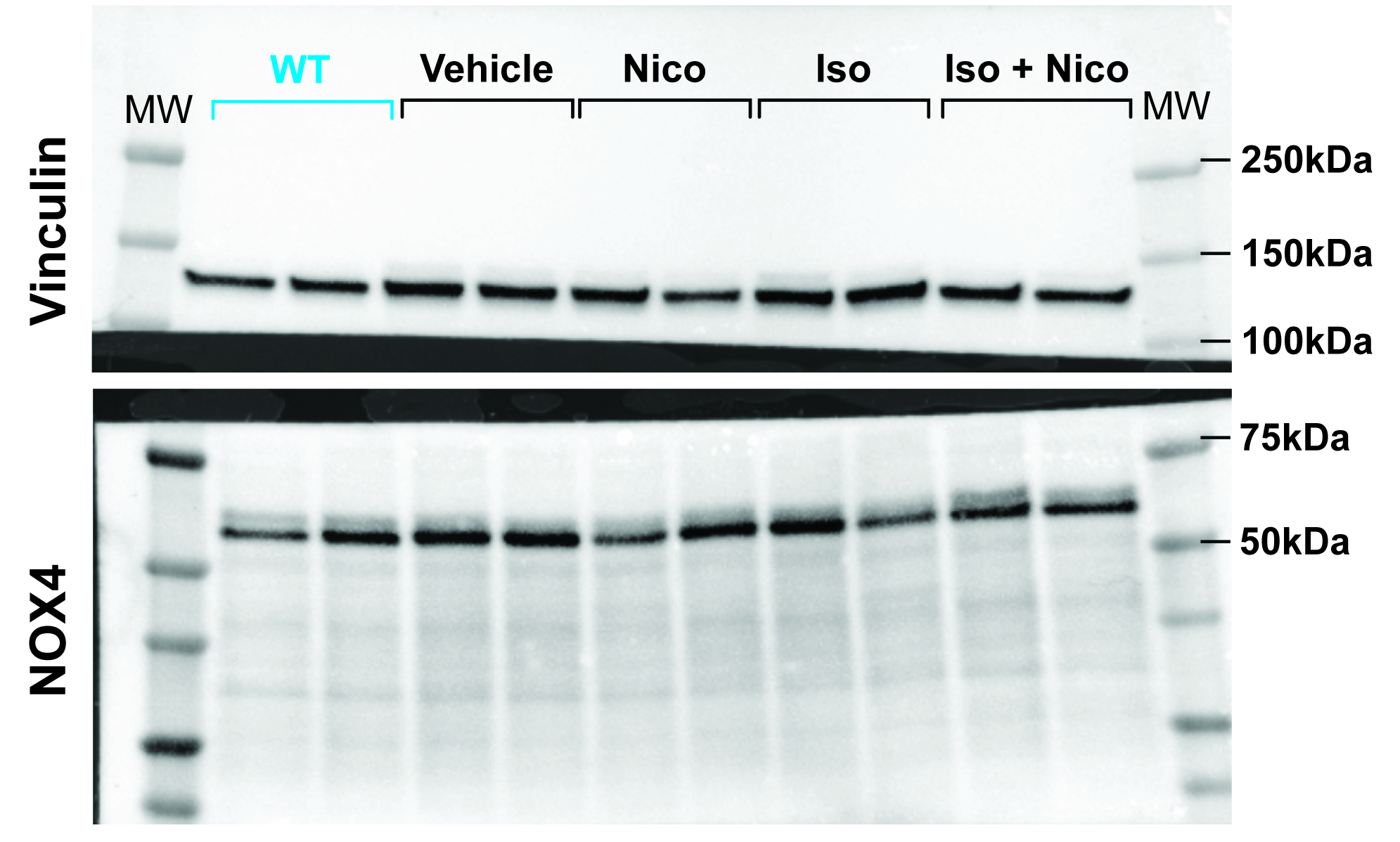

Supplement: Supplementary file 4 — Additional file 4.This supplemental figure shows the uncropped original blots for vinculin loading control and NADPH oxidase 4 (NOX4). [file 12872_2021_2112_MOESM4_ESM.tif]
